# Triphenyltin(IV) Carboxylates with Exceptionally High Cytotoxicity against Different Breast Cancer Cell Lines

**DOI:** 10.3390/biom13040595

**Published:** 2023-03-26

**Authors:** Ivana Predarska, Mohamad Saoud, Ibrahim Morgan, Peter Lönnecke, Goran N. Kaluđerović, Evamarie Hey-Hawkins

**Affiliations:** 1 Institute of Inorganic Chemistry, Faculty of Chemistry and Mineralogy, Leipzig University, Johannisallee 29, 04103 Leipzig, Germany; 2 Department of Engineering and Natural Sciences, University of Applied Sciences Merseburg, Eberhard Leibnitz-Str. 2, 06217 Merseburg, Germany; 3 Department of Bioorganic Chemistry, Leibniz Institute of Plant Biochemistry, Weinberg 3, 06120 Halle (Saale), Germany

**Keywords:** triphenyltin(IV), indomethacin, flurbiprofen, anticancer, breast cancer, NO production

## Abstract

Organotin(IV) carboxylates are a class of compounds explored as alternatives to platinum-containing chemotherapeutics due to propitious in vitro and in vivo results, and distinct mechanisms of action. In this study, triphenyltin(IV) derivatives of non-steroidal anti-inflammatory drugs (indomethacin (HIND) and flurbiprofen (HFBP)) are synthesized and characterized, namely [Ph_3_Sn(IND)] and [Ph_3_Sn(FBP)]. The crystal structure of [Ph_3_Sn(IND)] reveals penta-coordination of the central tin atom with almost perfect trigonal bipyramidal geometry with phenyl groups in the equatorial positions and two axially located oxygen atoms belonging to two distinct carboxylato (IND) ligands leading to formation of a coordination polymer with bridging carboxylato ligands. Employing MTT and CV probes, the antiproliferative effects of both organotin(IV) complexes, indomethacin, and flurbiprofen were evaluated on different breast carcinoma cells (BT-474, MDA-MB-468, MCF-7 and HCC1937). [Ph_3_Sn(IND)] and [Ph_3_Sn(FBP)], unlike the inactive ligand precursors, were found extremely active towards all examined cell lines, demonstrating IC_50_ concentrations in the range of 0.076–0.200 µM. Flow cytometry was employed to examine the mode of action showing that neither apoptotic nor autophagic mechanisms were triggered within the first 48 h of treatment. However, both tin(IV) complexes inhibited cell proliferation potentially related to the dramatic reduction in NO production, resulting from downregulation of nitric oxide synthase (iNOS) enzyme expression.

## 1. Introduction

Given that carcinoma is a main cause of death and a significant obstacle to raising life expectancy globally [1,2,3], it is not a surprise that the prime objective of contemporary medicinal chemistry is the creation of novel anticancer medications. After the unintentional finding of cisplatin in the 1960s [4] and the success it had treating several solid tumors types [5,6], numerous platinum-based compounds have undergone substantial research as potential chemotherapeutics [7,8,9]. However, the drawbacks related to using platinum-based therapies in clinical settings, such as systemic toxicity as well as innate and/or acquired resistance [10,11], have motivated scientists to expand the investigation also on other possible metal-based chemotherapeutic alternatives potentially exhibiting distinct modes of action. Many different metals, such as Ti, Fe, Ru, Os, Co, Rh, Ir, Pd, Cu, Au, Ga, Ge, and Sn, have spurred considerable attention in this context [12,13,14,15,16]. Among them, organotin(IV) complexes have appeared as very noteworthy non-platinum metallodrugs investigated by many research groups in the past four decades [17,18,19,20]. This is due to their reduced toxicity [17] and capacity to circumvent the medication resistance perceived for some commercially available metal-based pharmaceuticals [21]. The potential to induce apoptosis as well as to strongly interact with the deoxyribonucleic acid (DNA) are key for the chemotherapeutic capacity of organotin(IV) complexes [22]. The organic ligand is crucial for the bioactivity of these complexes; thus, Bu and Ph ligands result in more active compounds compared to those bearing Et and Me ligands [13,23]. The relative lipophilicity of the organotin moieties could be responsible for this effect [24]. The ligands’ nature is also closely related with the biological activity. Among the extensively studied thiolato [25,26,27,28,29,30], dithiocarbamato [31], and carboxylate [32,33,34,35,36,37,38,39] ligands, the last ones showed highest cytotoxic activity in vitro [24] and significant reduction in tumor growth in vivo [40,41].

Since it was discovered that the cyclooxygenase (COX) enzyme, particularly the isozyme COX-2, contributes to tumor generation and angiogenesis [42] and is overexpressed in certain tumor cells, such as stomach, colorectal, pancreas, bladder, breast, skin and esophagus [43], both non-steroidal anti-inflammatory drugs (NSAIDs), which are non-selective COX inhibitors, as well as COX-2-selective inhibitors (COXIBs) have been exploited as adjuvant chemotherapeutic and/or chemopreventive agents [44,45]. Complexes of different metals with NSAIDs have been previously prepared and discovered to have significant in vitro anticancer activity [46,47,48,49,50,51,52,53]. Complexes of organotin(IV) with NSAIDs have also been investigated. Triphenyltin(IV) complexes of flufenamic and mefenamic acid, namely triphenyltin(IV) flufenamate and mefenamate, have shown high in vitro cytotoxicity towards A549 (lung carcinoma), T-24 (bladder carcinoma) and MCF-7 (breast carcinoma) cells [54]. Different organotin(IV) ibuprofenate compounds have been synthesized as well and screened in vitro for cytotoxicity against Caco-2 (colorectal adenocarcinoma), HCT-15 (colon adenocarcinoma) and DU145 (prostate carcinoma) cells. According to the findings, triphenyltin(IV) ibuprofenate is most active against the cell line Caco-2 [55]. Oxaprozin is another NSAID used as carboxylate ligand in different di- and triorganotin(IV) complexes whose antiproliferative effects have been examined in vitro on a plethora of carcinoma cells including human colorectal adenocarcinoma (HT-29), hepatocellular (HepG2), breast (MCF-7), and prostate (PC-3) cancer cells. These complexes have shown excellent cytotoxicity with IC_50_ values of 0.10–0.76 µM. Once again, triphenyltin(IV) oxaprozinate has demonstrated greatest cytotoxicity towards the cancer cell line MCF-7 [56]. Several organotin(IV) indomethacinates have also been prepared; tri-*n*-butyltin(IV) and triphenyltin(IV) indomethacinate exhibited cytotoxic activity against the SK-LU-1 lung adenocarcinoma and the cervical cancer HeLa cells [57]. Additionally, different organotin(IV) derivatives with racemic flurbiprofenate as ligand have been prepared. However, they have only been tested as antibacterial and antifungal agents, while their cytotoxic activity has not been evaluated [58].

**Figure 1:**
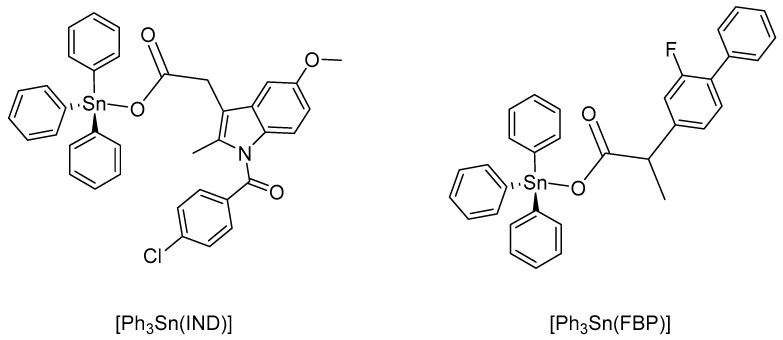
Organotin(IV) carboxylates: triphenyltin(IV) indomethacinate, [Ph_3_Sn(IND)], and triphenyltin(IV) flurbiprofenate, [Ph_3_Sn(FBP)].

Based on the latest cancer statistics, globally the most commonly diagnosed cancer and the number one reason for cancer-related death in women is female breast cancer [2]. Certain forms of breast cancer respond quite well to treatment. Yet, forms such as the triple-negative breast cancer (TNBC, estrogen-, progesterone- and HER-2-negative (ER−,PR−,HER2−)), are exceedingly difficult to medicate because of the disease’s complexity and absence of distinct molecular targets [59]. We have previously reported platinum(IV) complexes with different NSAIDs that proved to be highly effective towards different breast cancer cells. The cisplatin-indomethacinate (IND) conjugate was even able to overcome resistance expressed towards cisplatin by MDA-MB-231 breast carcinoma cells [60]. The racemic cisplatin-flurbiprofenate (FBP) conjugate showed even higher cytotoxic potency against four different breast cancer cell lines (MCF-7, HCC-1937, MDA-MB-468, BT-474) [61]. In order to assess how the metal ion affects the biological activity, we have also prepared two triphenyltin(IV) complexes with indomethacin and racemic flurbiprofen (corresponding carboxylate anions), namely [Ph_3_Sn(IND)] and [Ph_3_Sn(FBP)] (Figure 1). The two compounds have been previously reported [57,58]. In this study, however, these complexes were synthesized using different precursors; furthermore, a single crystal structure analysis of [Ph_3_Sn(IND)] is reported and the biological activity of both triphenyltin(IV) complexes was evaluated towards four cell lines of human breast carcinoma, namely BT-474, MCF-7, MDA-MB-468, and HCC1937, using colorimetric MTT- and CV-based cell viability assays. Furthermore, flow cytometry has been employed in order to understand the mechanism of the drug-induced cytotoxicity.

## 2. Materials and Methods

Reactions were performed implementing standard Schlenk techniques to ensure nitrogen atmosphere. Chemicals (triphenyltin(IV) chloride (Sigma-Aldrich Chemie GmbH, Steinheim, Germany), indomethacin (TCI), and racemic flurbiprofen (Biozol)) were used as purchased. Triethylamine was distilled from KOH and kept over 4 Å molecular sieves previously activated at 150 °C. Toluene was obtained from an MBraun Solvent Purification System (MBraun SPS-800) and kept over 4 Å active molecular sieves. A Heraeus VARIO EL oven was used for carrying out elemental analyses. NMR spectra were recorded on a Bruker AVANCE DRX 400 spectrometer. Tetramethylsilane (TMS) was used as internal standard to reference the NMR spectra; chemical shifts are reported in parts per million; ^1^H (400.13 MHz), ^13^C (100.61 MHz) and ^119^Sn (149.21 MHz). Mass spectra (HR-ESI-MS) were obtained with an FT-ICR-MS Bruker Daltonics ESI mass spectrometer (APEX II, 7 T). X-ray data from single crystals were collected with a Gemini-CCD diffractometer (Rigaku Oxford Diffraction, Oxford, UK).

### 2.1. Synthesis and Stability of Complexes

A stoichiometric amount of indomethacin or racemic flurbiprofen (0.45 g or 0.32 g, respectively), dissolved in 10 mL toluene, was added to a solution of Ph_3_SnCl (0.5 g, 1.3 mmol) in 10 mL toluene. After 20 min stirring, NEt_3_ (0.18 mL, 1.3 mmol, 1 eq.) was slowly (10 min) added to the solution and the resulting solution was stirred at room temperature overnight. The formed precipitate, (Et_3_NH)Cl, was filtered off. The solvent was evaporated and the solid residue was recrystallized from a 3:1 mixture of chloroform and methanol. For detailed characterization (multinuclear NMR, X-ray crystallographic data and HR-ESI-MS), see SI, Figures S1–S10. Spectroscopic data of [Ph_3_Sn(IND)] and [Ph_3_Sn(FBP)] are in agreement with those previously reported [57,58].

[Ph_3_Sn(IND)]: White solid; yield: 0.76 g (83%). Anal. calcd. for C_37_H_30_ClNO_4_Sn: C, 62.88; H, 4.28; N, 1.98%. Found: C, 62.46; H, 4.18; N, 1.93%.

[Ph_3_Sn(FBP)]: White solid; yield: 0.69 g (88%). Anal. calcd. for C_33_H_27_FO_2_Sn: C, 66.81; H, 4.59%. Found: C, 66.55; H, 4.29%.

Stability of complexes [Ph_3_Sn(IND)] and [Ph_3_Sn(FBP)] was evaluated in water-containing DMSO, employing time-resolved (0, 1, 3, 6, 12, 24, 48 and 72 h) ^1^H NMR spectroscopy.

### 2.2. Data Collection and Structure Refinement of [Ph_3_Sn(IND)]

The X-ray data from a single crystal of [Ph_3_Sn(IND)] were collected with a Gemini-CCD diffractometer (Rigaku Oxford Diffraction, Oxford, UK) employing ω-scan rotation and Mo-K_α_ radiation (λ = 0.71073 Å). Data reduction and empirical absorption correction were carried out using CrysAlisPro [62] with SCALE3 ABSPACK program for the later. For solving the structure, SHELXT-2018 [63] with dual-space method were employed, while structure refinement was performed with SHELXL-2018 [64] using full-matrix least-squares routines against F^2^. Anisotropic refinement was carried out for all non-hydrogen atoms, whereas hydrogen atoms were calculated on idealized positions. The C_19_H_15_ClNO_4_ substituent is disordered on two positions with a ratio of 0.814(5):0.186(5). All chloroform solvent molecules are disordered as well. DIAMOND-4 [65] was used to generate all structural figures. Crystallographic details are listed in Table S1 in the SI.

### 2.3. In Vitro Studies

The four cell lines of human breast carcinoma utilized in this study were acquired from ATCC, Manassas, USA. Cell viability assays (MTT (3-(4,5-dimethylthiazol-2-yl)-2,5-diphenyltetrazolium bromide) and CV (crystal violet)) were bought from Sigma-Aldrich, Taufkirchen, Germany. Multi-well plates, culture flasks and additional plastics for cell culturing were bought from TPP, Trasadingen, Switzerland and Greiner Bio-One GmbH, Frickenhausen, Germany. Fetal calf serum (FCS), RPMI cell culturing medium, L-glutamine, trypsin/ethylenediaminetetraacetic acid (EDTA) and phosphate-buffered saline (PBS) were all obtained from Capricorn Scientific GmbH, Ebsdorfergrund, Germany. The reagents for fluorescence-activated cell sorting (FACS) were obtained from several suppliers: Thermo Fisher Scientific: annexin V/propidium iodide (AnnV/PI) and 4-amino-5-methylamino-2′,7′-difluorofluorescein diacetate (DAF-FM); BD horizon: dihydrorhodamine (DHR) and carboxyfluorescein succinimidyl (CFSE); Sigma Aldrich: acridine orange (AO) and 4′,6-diamidino-2-phenylindole (DAPI); R & D scientific: ApoStat.

### 2.4. Cell Lines, General Conditions and IC_50_ Determination

Cytotoxic effects of the NSAIDs used as ligands, indomethacin and flurbiprofen, and organotin(IV) compounds [Ph_3_Sn(IND)] and (Ph_3_Sn(FBP)] were evaluated using colorimetric MTT- and CV-based cell viability assays against four cell lines from different human breast carcinoma. BT-474 and MCF-7 are estrogen-positive cells, while MDA-MB-468 and HCC1937 are triple-negative cells [66]. Cells’ cultivation was carried out in T-75 flasks using RPMI 1640 medium supplied with 10% FCS inactivated by heat, L-glutamine (2 mM) and 1% penicillin/streptomycin, at 37 °C, in a humidified environment with 5% CO_2_ until subconfluency ~ 80% was reached. Cell passaging and seeding was carried out after washing of adherent cells using PBS and detaching them applying trypsin/EDTA solution (0.05%) in PBS.

Utilizing the aforementioned cell growth medium, cells were seeded in plates with 96 wells, at a 6000/100 µL/well density. Before treatment with the investigated compounds, cells were given 24 h to adhere. Stock solutions of cisplatin, indomethacin, flurbiprofen, [Ph_3_Sn(IND)], and [Ph_3_Sn(FBP)] were made in DMSO and subsequently diluted in growth medium to reach the following concentrations: 300, 100, 30, 10, 3, 1, and 0.1 μM for cisplatin, 100, 50, 25, 12.5, 6.25, 3.125, and 1.6 μM for the NSAIDs and 10, 5, 1, 0.5, 0.1, 0.01, and 0.001 μM for [Ph_3_Sn(IND)] and [Ph_3_Sn(FBP)]. A positive control of digitonin (100 μM) was included in each 96-well plate. Three independent biological replicates and four technical replicates were conducted. After 72 h treatment, cell viability was assessed. For the MTT assay, cells were initially rinsed with PBS and then exposed for 1 h to the working solution containing 0.5 mg mL^−1^ MTT in culture medium. The MTT solution was then removed, the formazan formed was dissolved in DMSO and its absorbance was measured at 570 nm and 670 nm employing a multi-well plate reader SpectraMax M5 (Molecular Devices, San Jose, CA, USA). For the CV assay, cells were first washed with PBS, fixated with 4% paraformaldehyde (PFA), dried after removal of the PFA solution and only then stained for 20 min with a 10% crystal violet solution. After discarding the staining solution, stained cells were supplied with acetic acid (33% in aqua bidest.) and the absorbance was recorded by using the previously described method at the same wavelengths [67]. Cell viability is expressed as a percentage of untreated cells with the mean value being computed using a four-parametric logistic function [68]. SigmaPlot 14.0 and Microsoft Excel 2013 programs were used for data analysis and IC_50_ value calculation.

### 2.5. Flow Cytometry

BT-474 cells were seeded in 6-well plates, with a density of 150,000 cells/well. Cells were allowed to adhere overnight, after which they were treated with IC_50_ value concentrations of [Ph_3_Sn(IND)], [Ph_3_Sn(FBP)] and cisplatin and subjected to flow cytometry (BD FACSAria III) assessment. For that, several staining procedures were employed including (1) AnnV/PI to identify cells undergoing apoptosis, (2) ApoStat to detect caspase activity, (3) AO to monitor autophagy induction, (4) DHR to measure reactive oxygen species/reactive nitrogen species (ROS/RNS), (5) DAF-FM to detect intracellular NO, and (6) CFSE to track the impact on cellular proliferation.

AnnV/PI, ApoStat, AO and DAF-FM staining was carried out according to the manufacturer’s recommendations after 48-h-long exposure to the examined compounds, trypsinization and washing with PBS. AnnV/PI (5% AnnV, 2% PI in PBS) [69] and AO (1 µg mL^−1^ PBS) staining solutions were applied for 15 min at room temperature or at 37 °C, respectively, in an environment with 5% CO_2_. For caspase activity determination, 30 min exposure to the ApoStat (1% ApoStat, 5% FCS in PBS) stain at 37 °C with 5% CO_2_ was applied. For NO production analysis, cells were dyed with DAF-FM dye (5 μM DAF-FM, 10% FCS in RPMI) for 1 h at 37 °C with 5% CO_2_. To neutralize the stain, the cells were incubated in serum-free medium for 15 min. Finally, cells’ detachment was performed and flow cytometry examination was conducted through measurement of fluorescein isothiocyanate (FITC) fluorescence.

For ROS/RNS production analysis and cell proliferation investigations, cells were first dyed with the appropriate stain solution before being subjected to 48 h treatment with the experimental agents and cisplatin. DHR (1 μM DHR, 0.1% FCS in PBS) and CFSE (1 μM CFSE, 0.1% FCS in PBS) [70] stains were applied overnight at 37 °C with 5% CO_2_. After dyeing and 48 h treatment, trypsin-EDTA was used to detach the cells, which were subsequently rinsed with PBS and finally analyzed by flow cytometry.

## 3. Results and Discussion

### 3.1. Synthesis, Characterization, and Stability of Organotin(IV) Carboxylates

The two organotin(IV) complexes, triphenyltin(IV) indomethacinate [Ph_3_Sn(IND)], and triphenyltin(IV) flurbiprofenate [Ph_3_Sn(FBP)], were prepared in a reaction of the corresponding NSAID (HIND or HFBP) deprotonated with NEt_3_ and an equimolar amount of Ph_3_SnCl. The preparation of both organotin(IV) compounds, starting from triphenyltin(IV) chloride, resulted in higher yields and easier purification of the obtained compounds then previously reported procedures involving Ph_3_SnOH as starting material [57,58]. Elemental analysis was employed to verify purity of the produced complexes. Further characterization of the compounds was also carried out by multinuclear NMR spectroscopy (^1^H, ^13^C, ^119^Sn) and mass spectrometry. The obtained results, presented in the SI (Figures S1–S10), are in agreement with the reported ones [57,58].

The complexes [Ph_3_Sn(IND)] and [Ph_3_Sn(FBP)] are stable in water-containing DMSO solution during a 72-h period, as shown by time-resolved ^1^H NMR spectroscopy. No ligand exchange or complex degradation was observed during this time (Figures S11 and S12).

**Figure 2:**
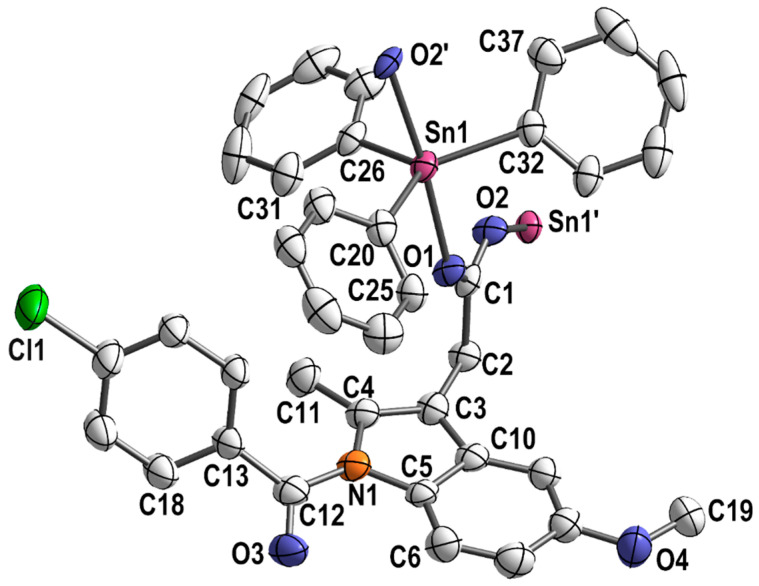
Section of the polymeric structure of [Ph_3_Sn(IND)]; hydrogen atoms and solvent molecules are omitted for clarity; thermal ellipsoids are set at 50% probability level.

**Figure 3:**
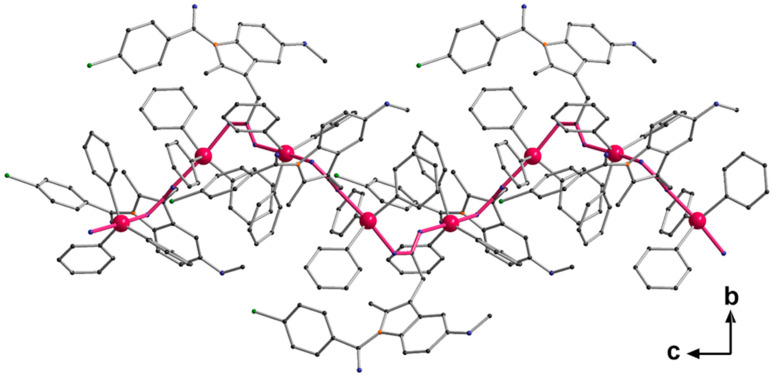
Helical chain structure of [Ph_3_Sn(IND)], propagation via O → Sn coordination. Bonds forming the basis of the helix are highlighted in pink.

Single crystals adequate for X-ray diffraction studies were formed slowly diffusing methanol into a CHCl_3_ solution of [Ph_3_Sn(IND)]. [Ph_3_Sn(IND)] crystallizes with sixteen monomeric units per unit cell, in the tetragonal space group *I*4_1_/*a.* The trigonal-planar SnPh_3_ moieties are connected by bridging carboxylato ligands in the axial positions (Figure 2) forming polymeric helical chains (Figure 3). Consequently, with equatorial phenyl groups and two axial oxygen atoms belonging to two distinct carboxylato (IND) ligands, the penta-coordinated central tin atom displays an almost perfect trigonal bipyramidal geometry. Thus, the C–Sn1–O, C–Sn1–C (C20–Sn1–C26, C20–Sn1–C32 and C26–Sn1–C32), and O1–Sn–O2′ angles are close to 90, 120 and 180°, respectively. The Sn–O (Sn1–O1 2.182(7), Sn1–O2′ 2.274(7) Å) and Sn–C bond lengths (Sn1–C26 2.108(5), Sn1–C20 2.129(5), Sn1–C32 2.129(5) Å) are comparable to ones reported for analogous triorganotin(IV) carboxylate complexes [71,72,73,74,75,76]. The carboxylato ligand bridges two tin atoms which are symmetry-independent resulting in distinct Sn–O bond lengths (Table 1).

### 3.2. Cytotoxic Activity

Following a 72 h treatment, the in vitro cytotoxicity of [Ph_3_Sn(IND)] and [Ph_3_Sn(FBP)] was evaluated in a variety of breast carcinoma cells, namely triple-positive (ER+,PR+,HER2+) BT-474 causing invasive ductal breast cancer, COX-1 expressing MCF-7 (ER+,PR+,HER2−), COX-2 expressing MDA-MB-468 (ER−,PR−,HER2−), and HCC1937 cancer cells with BRCA1 mutation (ER−,PR−,HER2−). Investigations were made with two distinct cell viability assays, MTT and CV. MTT is an indirect method for cell viability determination which utilizes the capacity of living cells to catalyze tetrazolinum salt reduction in MTT to formazan [77]. This is the result of mitochondrial dehydrogenase activity taking place in the mitochondria of viable cells. Consequently, substances that alter cellular metabolism through elevation of the level of reduced nicotinamide adenine dinucleotide phosphate (NADPH) or the activity of lactate dehydrogenase (LDH) may have a considerable impact on the results of the MTT experiment [78,79,80,81]. Therefore, for higher reliability of the results, also the direct, non-enzymatic CV assay was employed. The cytotoxicity of the organotin(IV) complexes was compared to that of the two NSAIDs utilized as ligands, indomethacin and flurbiprofen, as well as cisplatin which is used as standard clinical therapy. The decrease in cell viability in the selected cell lines upon treatment with increasing concentrations of [Ph_3_Sn(IND)], [Ph_3_Sn(FBP)] and cisplatin determined by MTT and CV assays is presented in the SI (Figures S13–S16). The IC_50_ values are reported in Table 2.

As expected, none of the four tumor cell lines was susceptible to the COX inhibitors alone (>100 μM). The cytotoxic potential of both organotin(IV) carboxylates, on the contrary, was significantly higher compared to cisplatin’s activity, exhibiting nanomolar IC_50_ values in all cases. Both complexes [Ph_3_Sn(IND)] and [Ph_3_Sn(FBP)] had a comparable effect on all four cell lines. With regard to cisplatin, their activity was much higher, resulting in at least 24 times lower IC_50_ values towards the MDA-MB-468 cell line and up to 366 times lower IC_50_ values towards the BT-474 cell line. Similarly high activity was previously proven for [Ph_3_Sn(IND)] against HeLa cervical cancer cells and SKLU-1 lung adenocarcinoma cells [57]. The investigated complexes showed much higher activity with respect to the structurally similar complex with an ibuprofenate ligand ([Ph_3_Sn(IBF)]) which has been found to be active against the Caco-2 colorectal adenocarcinoma cell line, but only remotely active or completely inactive against the DU145 (prostate carcinoma) cells and the HCT-15 (colon adenocarcinoma) cells, respectively [55]. In our previous study, flurbiprofen was used as an axial ligand of a cisplatin-based platinum(IV) conjugate and the cytotoxic activity of this *cis*,*trans*,*cis*-[PtCl_2_(FBP)_2_(NH_3_)_2_] complex was assessed against the same four breast carcinoma cell lines used in the present study [61]. The obtained results show very similar cytotoxic activity for [Ph_3_Sn(FBP)] and the cisplatin-flurbiprofenate complex suggesting that both NSAID-metal complexes with tin(IV) and platinum(IV) are highly active. Since the COX inhibitory potential of the prepared metal complexes was not assessed in these studies, the mechanism underlying the increase in cytotoxic efficacy following NSAID conjugation is unclear. The findings from the CV and MTT assays for [Ph_3_Sn(IND)] and [Ph_3_Sn(FBP)] are in very good agreement with each other, while for cisplatin towards the BT-474, HCC1937 and MDA-MB-468 cells, some discrepancies were identified. This suggests that the investigated organotin(IV) carboxylates have different mechanism of action than cisplatin, not affecting the cell metabolism pathways.

### 3.3. Mode of Cytotoxic Activity

Flow cytometry was employed in order to comprehend the mechanism underlying the drug-induced cytotoxicity. In this method, a specific type of cell in a heterogeneous environment is recognized and physically separated using fluorescently labeled target-specific antibodies. This enables analysis of the nucleic acid material, presence of characteristic proteins, as well as phenotype-specific metabolic content and, therefore, valuable information on cell proliferation, apoptosis and autophagy, ROS/RNS, and NO production, etc., can be acquired. These data allow for conclusions regarding the drug’s mechanism of action to be drawn. In the present investigation, BT-474 cells underwent treatment with IC_50_ doses of [Ph_3_Sn(IND)], [Ph_3_Sn(FBP)] and cisplatin before undergoing a variety of FACS assessments. This cell line was chosen for the mechanistic studies because of the high cytotoxicity of the investigated compounds against it, which is also the greatest advancement in comparison to cisplatin.

**Figure 4:**
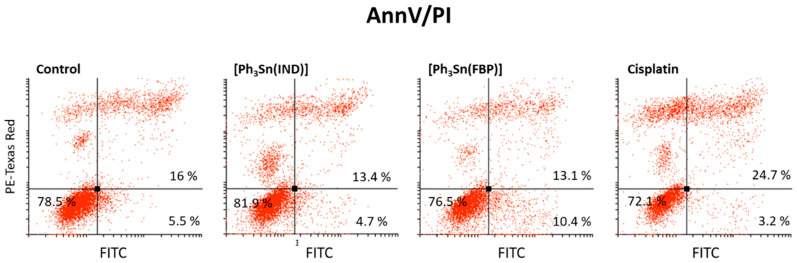
Capacity of organotin(IV) complexes ([Ph_3_Sn(IND)] and [Ph_3_Sn(FBP)]) and cisplatin to induce apoptotic cell death—early and late apoptotic cells (after 48 h treatment).

In order to determine whether [Ph_3_Sn(IND)] and [Ph_3_Sn(FBP)] induce apoptotic cell death, AnnV/PI staining was used. This method relies on the relocation of the membrane phospholipid phosphatidylserine (PS) from the internal to the external side of the membrane, causing a change of the plasma membrane asymmetry, which is a key hallmark of apoptosis. Because of its strong affinity for PS, the annexin V dye acts as a sensor for cells undergoing apoptosis including early apoptotic cells [82]. As the apoptotic process progresses, the intactness of the membrane is lost and it becomes permeable for the second dye, PI. Therefore, cells that are both AnnV- and PI-positive, are in late apoptosis or dead already. Results presented in Figure 4 show that after 48 h treatment with [Ph_3_Sn(IND)] and [Ph_3_Sn(FBP)], an apoptotic process is not yet ongoing and the number of surviving cells is not significantly influenced, while cisplatin treatment leads to a slight elevation of late apoptotic BT-474 cells population.

**Figure 5:**
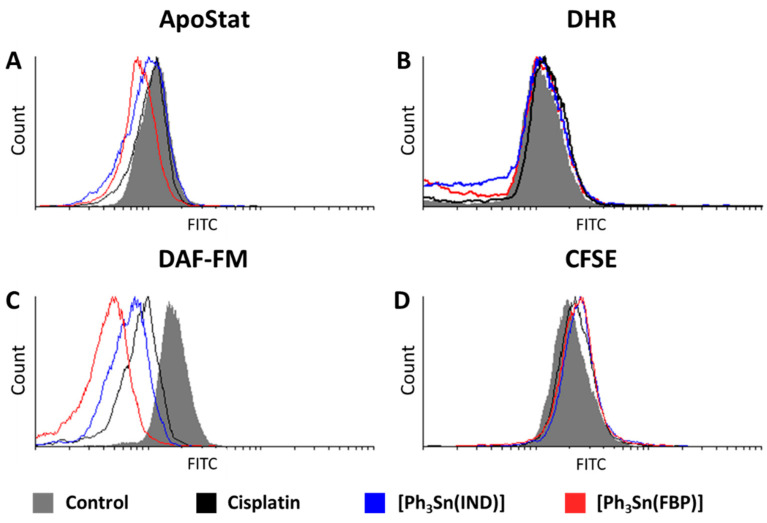
Effect of organotin(IV) complexes ([Ph_3_Sn(IND)] and [Ph_3_Sn(FBP)]) and cisplatin on BT-474 cells: (**A**) caspase activation, (**B**) ROS/RNS production, (**C**) NO production, and (**D**) cell proliferation potential.

Identification of activated caspases is another marker of apoptosis. These cytosolic cysteine proteases are implicated in the onset as well as execution of apoptosis. Therefore, detection of this enzyme’s activity indicates the presence of apoptotic cells. This can be probed utilizing the ApoStat assay. Figure 5A reveals that [Ph_3_Sn(IND)] and [Ph_3_Sn(FBP)] do not activate, but rather inhibit the activity of these enzymes, whereas cisplatin has no effect on the caspase activity, although previous reports link cisplatin-induced cell death with caspase-activated apoptosis [61,83,84,85,86,87]. These results, however, are in agreement with the ones obtained with the AnnV/PI experiments and confirm that apoptotic mechanisms in BT-474 cells could not be detected within the 48 h of treatment.

Lack of impact was also the outcome of the DHR staining investigation conducted with the aim to assess the influence of [Ph_3_Sn(IND)] and [Ph_3_Sn(FBP)] and cisplatin on the production of ROS and RNS. Although increased ROS/RNS production has been previously related to cytotoxicity brought on by cisplatin [83,88,89] as well as cisplatin-NSAID conjugates [61], in BT-474 cells treated with cisplatin or [Ph_3_Sn(IND)] and [Ph_3_Sn(FBP)], only slightly elevated ROS/RNS production could be detected (Figure 5B). Thus, on breast BT-474 cells the tested complexes did not induce cell death by orchestrating antioxidant systems [90].

Interestingly, all three compounds have a most prominent effect on the generation of nitric oxide resulting in a significant decrease in NO. This effect, as displayed in Figure 5C, is most pronounced for the tin(IV)-based compounds with [Ph_3_Sn(FBP)] being the more potent NO suppressor, while cisplatin causes the least notable inhibition. Nitric oxide is a bioactive molecule having a profound impact on numerous physiological and pathological processes [91]. In cancer biology, the role of NO is controversial as it can exert both carcinogenic or anticancer effects, depending on the location, time, and its concentration [92,93,94,95]. NO is produced enzymatically from nitric oxide synthase (NOS). Despite the existence of three isoforms of this enzyme (neuronal, inducible, and endothelial), the inducible form (iNOS) has the most compelling association with tumor progression and metastasis [92]. Namely, elevated iNOS expression has been reported for different cancers [96,97,98,99], including breast cancer [100,101,102,103], where iNOS overexpression has been linked with tumor aggressiveness and poor prognosis for the patients. Furthermore, Chang et al. [104] have shown that treatment with iNOS inhibitors can suppress tumor cell proliferation as well as cancer stem cells’ capacity for self-renewal and migration, hence lowering tumor initiation, growth, and the incidence of lung metastases from breast cancer. Considering the fact that our findings suggest that the metal complexes cause inhibition of cell proliferation (evaluated with a CFSE assay; Figure 5D) in BT 474 cells, it can be assumed that the suppression of NO production correlates to reduced cell proliferation, possibly through decreasing iNOS gene expression. A previous study reports that flurbiprofen has the ability to inhibit iNOS expression in RAW 264.7 macrophages [105]. Furthermore, indomethacin has been found to decrease iNOS gene expression and reduce tumor growth in vivo in breast tumor-bearing mice [106]. These findings regarding our ligand precursors, indomethacin and flurbiprofen, further corroborate our conclusions that the cytotoxic capacity of [Ph_3_Sn(IND)] and [Ph_3_Sn(FBP)] could be due to iNOS suppression-mediated reduction in NO production.

**Figure 6:**
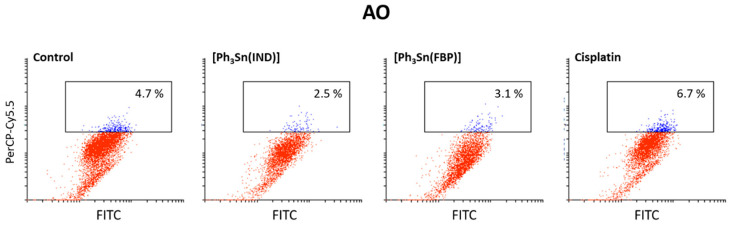
Autophagy induction in BT-474 cells upon 48 h treatment with organotin(IV) complexes ([Ph_3_Sn(IND)] and [Ph_3_Sn(FBP)]) and cisplatin.

Finally, an acridine orange (AO) assay was used to investigate if [Ph_3_Sn(IND)] and [Ph_3_Sn(FBP)] as well as cisplatin can trigger autophagy in BT-474 cells. This controlled lysosomal mechanism is engaged in the breakdown and reuse of cytoplasmic components. Amino and fatty acids, which are being created during this process, can be utilized to produce proteins or energy, which are critical for a cell’s ability to survive in a starvation situation. However, autophagy can also result in cell death upon exposure to chemical therapy [107]. According to our findings (Figure 6), cisplatin, [Ph_3_Sn(IND)] and [Ph_3_Sn(FBP)] did not have any significant influence on autophagy after 48 h treatment, thus neither cytoprotective nor cytotoxic effect of autophagy [108] can be expected upon treatment of B-474 cells with the investigated complexes.

## 4. Conclusions

In this study, two triphenyltin(IV) carboxylates with NSAIDs as ligands, [Ph_3_Sn(IND)] and [Ph_3_Sn(FBP)], were prepared and evaluated for their in vitro antiproliferative effect towards four different cell lines of breast carcinoma. The ligand precursors, indomethacin and flurbiprofen, exhibited no impact on the examined cancer cells’ proliferation. On the other hand, the organotin(IV) complexes demonstrated IC_50_ values at nanomolar concentrations in the range of 0.076–0.200 µM. This superior cytotoxicity, demonstrated against all cell lines involved, represents a significant enhancement in comparison to the effect of cisplatin. Various biological experiments used to investigate these compounds’ mode of action suggested that neither apoptotic nor autophagy mechanisms were activated within the 48 h treatment. Ho wever, slightly elevated ROS/RNS production as well as inhibition of the cell proliferation were confirmed for these compounds; the latter might be related to the massive suppression of NO production. Gene expression experiments could be additionally performed to evaluate if the extensive NO production suppression is caused by inhibition of iNOS expression.

## Figures and Tables

**Table 1:** Selected Bond Lengths [Å] and Angles [°] for [Ph_3_Sn(IND)].

Bond Lengths [Å]		Bond Angles [°]	
Sn1–O1	2.182(7)	C20–Sn1–C26	116.1(2)
Sn1–O2′	2.274(7)	C26–Sn1–C32	121.1(2)
Sn1–C26	2.108(5)	C20–Sn1–C32	122.7(2)
Sn1–C20	2.129(5)	C–Sn1–O	83.9(2) to 95.3(3)
Sn1–C32	2.129(5)	O1–Sn1–O2′	174.6(5)
symmetry operation ‘ −y + 3/4,x + 1/4,z + 1/4			

**Table 2:** IC_50_ values (mean ± SD, [µM]) of synthesized complexes and cisplatin obtained with MTT and CV assays after 72 h treatment. IC_50_ values of the ligand precursors, indomethacin and flurbiprofen, are >100 μM as found by both MTT and CV assays after 72 h treatment.

		MDA-MB-468	HCC1937	MCF-7	BT-474
		IC_50_ [µM]			
[Ph_3_Sn(IND)]	MTT CV	0.11 ± 0.03 0.14 ± 0.02	0.15 ± 0.01 0.20 ± 0.02	0.13 ± 0.02 0.17 ± 0.01	0.10 ± 0.02 0.15 ± 0.02
[Ph_3_Sn(FBP)]	MTT CV	0.11 ± 0.01 0.12 ± 0.02	0.13 ± 0.01 0.17 ± 0.01	0.12 ± 0.03 0.16 ± 0.01	0.076 ± 0.003 0.16 ± 0.01
Cisplatin	MTT CV	0.60 ± 0.11 3.32 ± 0.19	4.26 ± 0.73 7.62 ± 0.90	32.00 ± 4.29 33.59 ± 4.83	70.30 ± 8.45 54.86 ± 6.03

## Data Availability

Data is contained within the article or Supplementary Materials.

## Supplementary Materials

The following supporting information can be downloaded at: https://www.mdpi.com/article/10.3390/biom13040595/s1, Figure S1: 1H NMR spectrum of [Ph3Sn(IND)] in CDCl3; Figure S2: 13C{1H} NMR spectrum of [Ph3Sn(IND)] in CDCl3; Figure S3: 119Sn{1H} NMR spectrum of [Ph3Sn(IND)] in CDCl3; Figures S4 and S5: HR-ESI-MS of [Ph3Sn(IND)] (positive mode, CH3OH); Figure S6: 1H NMR spectrum of [Ph3Sn(FBP)] in CDCl3; Figure S7: 13C{1H} NMR spectrum of [Ph3Sn(FBP)] in CDCl3; Figure S8: 119Sn{1H} NMR spectrum of [Ph3Sn(FBP)] in CDCl3; Figures S9 and S10: HR-ESI-MS of [Ph3Sn(FBP)] (positive mode, CH3OH); Figure S11:. Stability of [Ph3Sn(IND)2] in DMSO-d6 over 72 h; time-resolved 1H NMR spectra; Figure S12: Stability of [Ph3Sn(FBP)2] in DMSO-d6 over 72 h; time-resolved 1H NMR spectra; Figure S13: Cell viability of [Ph3Sn(IND)], [Ph3Sn(FBP)] and cisplatin determined by MTT and CV assays in MDA-MB-468 breast cancer cell line; Figure S14: Cell viability of [Ph3Sn(IND)], [Ph3Sn(FBP)] and cisplatin determined by MTT and CV assays in HCC1937 breast cancer cell line; Figure S15: Cell viability of [Ph3Sn(IND)], [Ph3Sn(FBP)] and cisplatin determined by MTT and CV assays in MCF-7 breast cancer cell line; Figure S16: Cell viability of [Ph3Sn(IND)], [Ph3Sn(FBP)] and cisplatin determined by MTT and CV assays in BT-474 breast cancer cell line; Table S1: Crystal data and structure refinement of [Ph_3_Sn(IND)].
