# Supplementary material for: Triphenyltin(IV) Carboxylates with Exceptionally High Cytotoxicity against Different Breast Cancer Cell Lines
Source: Biomolecules. 2023 Mar 26;13(4):595. doi: 10.3390/biom13040595 (PMC10136130; doi:10.3390/biom13040595)
Supplement: Supplementary file 1 [file biomolecules-13-00595-s001.zip › biomolecules-2291606-supplementary.pdf]

Triphenyltin(IV) Carboxylates with Exceptionally High Cytotoxicity against Different Breast Cancer Cell Lines

Ivana Predarska, Mohamad Saoud, Ibrahim Morgan, Peter Lönnecke, Goran N. Kaluđerović* and Evamarie Hey-Hawkins*

Electronic Supplementary Information

Table of Contents

Characterization of complexes 2

NMR Spectra of [Ph_3Sn(IND)] 2

Mass Spectrum of [Ph_3Sn(IND)] 4

X-ray Crystallography 6

NMR Spectra of [Ph_3Sn(FBP)] 7

Mass Spectra of [Ph_3Sn(FBP)] 10

Stability of complexes [Ph_3Sn(IND)] and [Ph_3Sn(FBP)] in DMSO 11

Cell viability of complexes 12

Characterization of complexes

NMR Spectra of [Ph_3Sn(IND)]

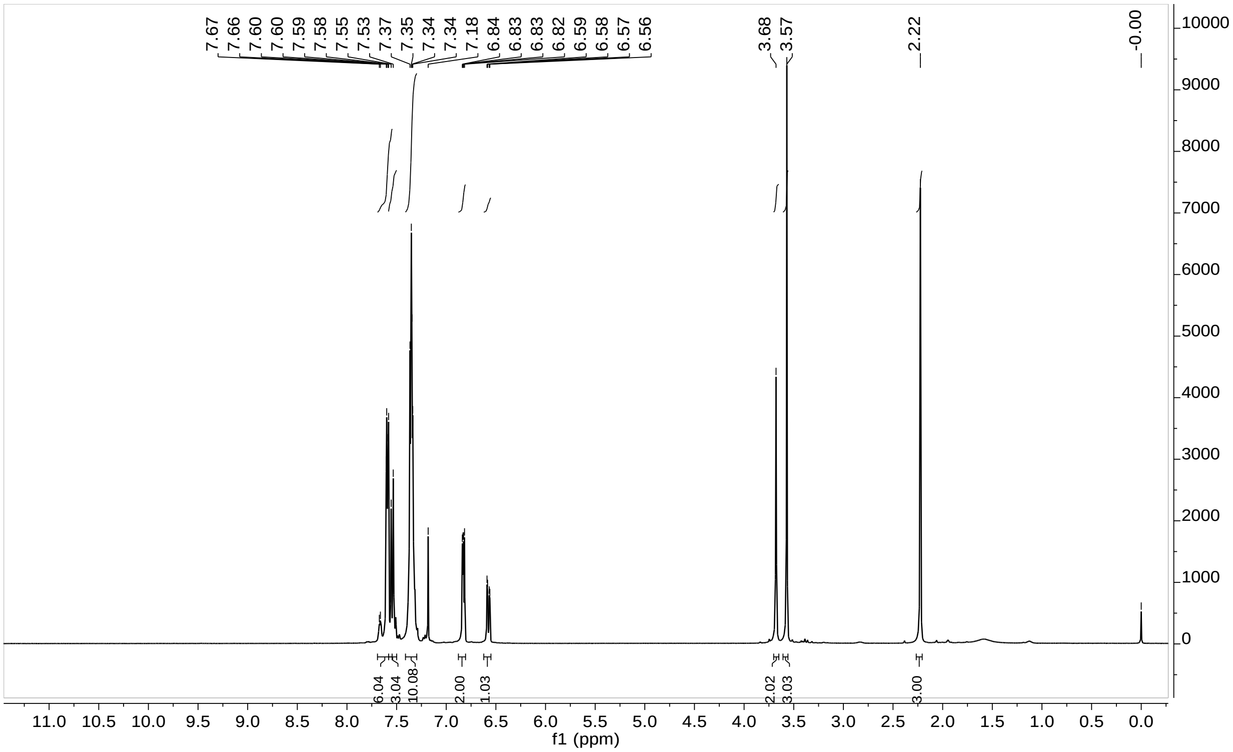

Figure S1. ^{1}H NMR spectrum of [Ph_3Sn(IND)] in $CDCl_3$.

^{1}H NMR ($CDCl_3$, ppm, 400 MHz): δ = 7.67 – 7.58 (m, br., 6H, CH_{aryl}), 7.54 (d, $^3J_{HH}$ = 8 Hz, 3H, CH_{aryl}), 7.37 – 7.34 (m, br., 10H, CH_{aryl}), 6.83 (dd, $^3J_{HH}$ = 8 Hz, $^4J_{HH}$ = 2 Hz 2H, CH_{aryl}), 6.57 (dd, $^3J_{HH}$ = 8 Hz, $^4J_{HH}$ = 2 Hz 1H, CH_{aryl}), 3.68 (s, 2H, CH_2), 3.75 (s, 3H, OCH_3), 2.22 (s, 3H, CH_3).

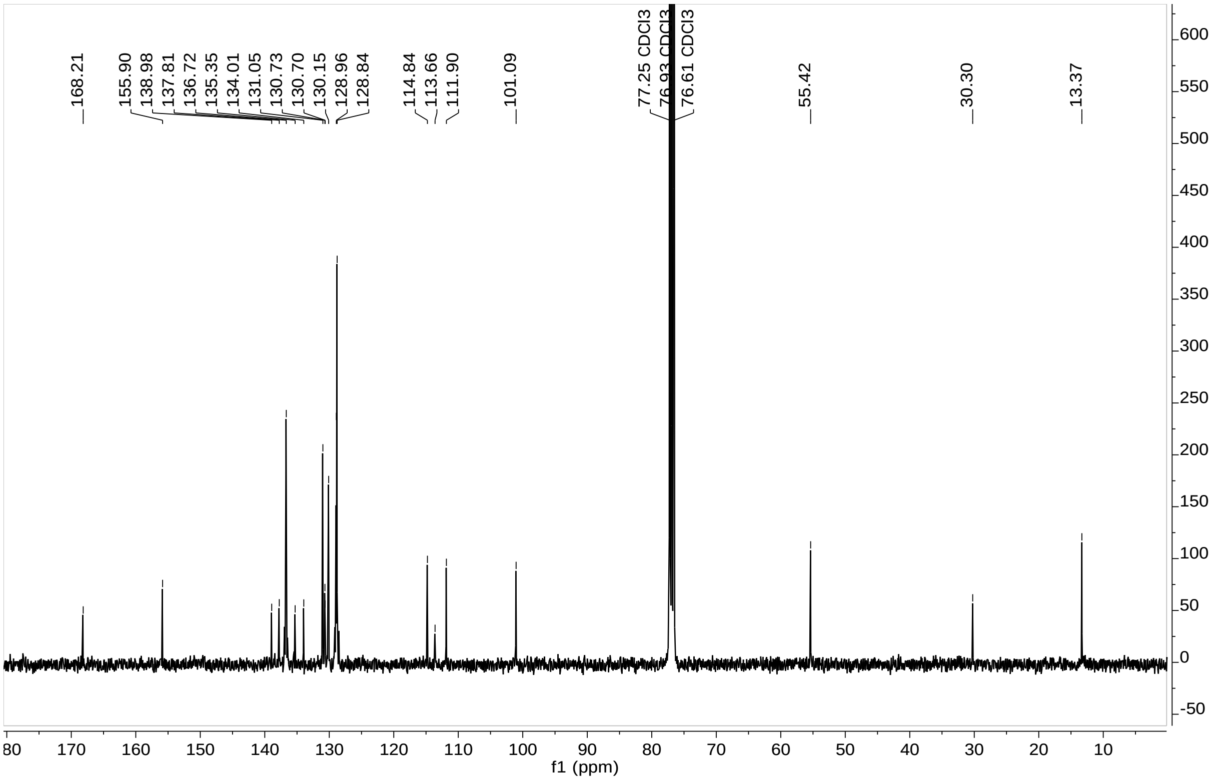

Figure S2. ^{13}C{^{1}H} NMR spectrum of [Ph_3Sn(IND)] in $CDCl_3$.

^{13}C{^{1}H} NMR ($CDCl_3$, ppm, 100.6 MHz): δ = 168.2 (qC, *C*O), 155.9 (qC, C_{aryl}), 138.9 (qC, C_{aryl}), 137.8 (qC, C_{aryl}), 136.7 (CH, C_{aryl}), 135.4 (qC, C_{aryl}), 134 (qC, C_{aryl}), 131 (CH, C_{aryl}), 130.7 (qC, C_{aryl}), 130.7 (qC, C_{aryl}), 130.2 (qC, C_{aryl}), 128.9 (CH, C_{aryl}), 128.8 (CH, C_{aryl}), 114.8 (qC, C_{aryl}), 113.7 (CH, C_{aryl}), 111.9 (CH, C_{aryl}), 101 (CH, C_{aryl}), 55.4 (CH_3, OCH_3), 30.3 (CH_2), 13.4 (CH_3).

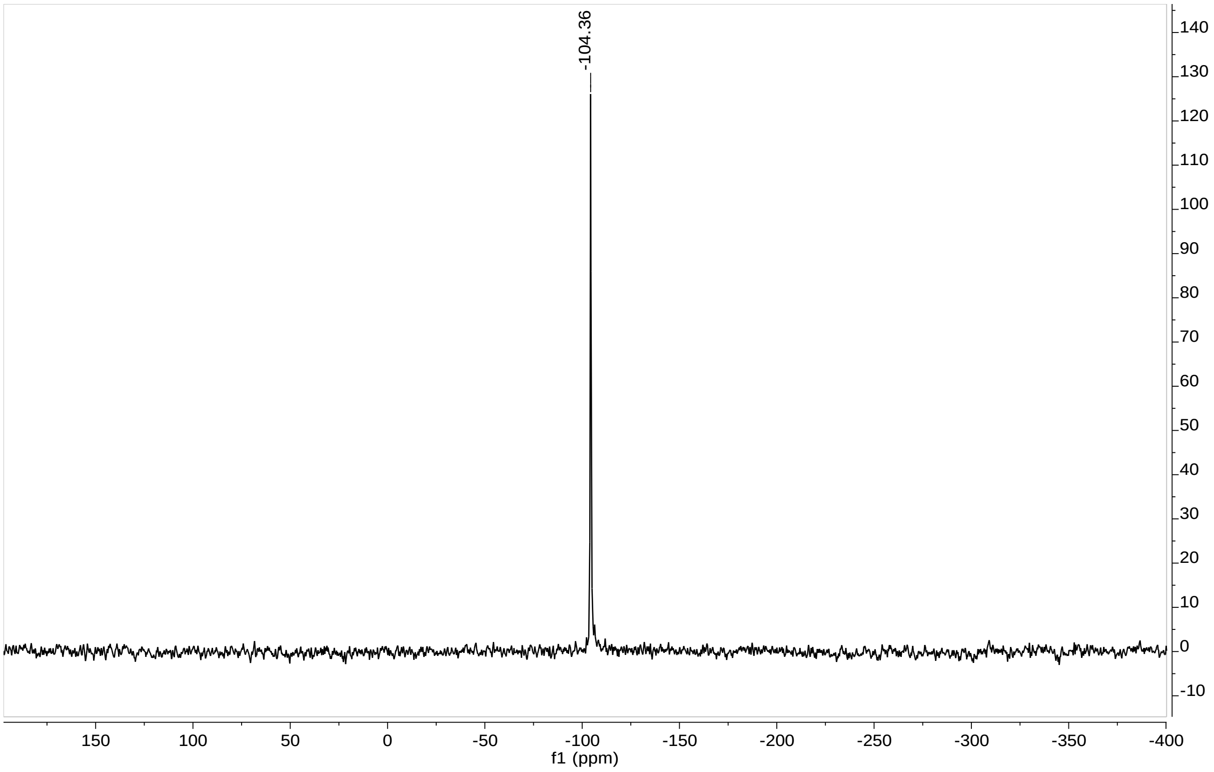

Figure S3. $^{119}Sn\{^1H\}$ NMR spectrum of [Ph_3Sn(IND)] in $CDCl_3$.

^{119}Sn{^{1}H} NMR ($CDCl_3$, ppm, 149.2 MHz): δ = -104.36.

Mass Spectrum of [Ph_3Sn(IND)]

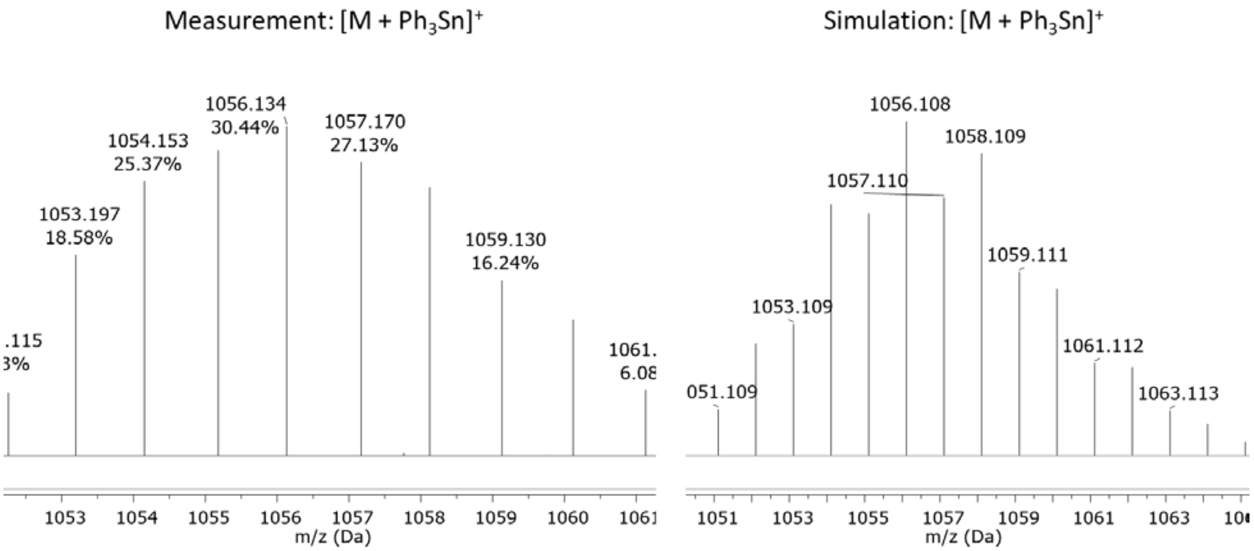

Figure S4. HR-ESI-MS (positive mode, CH_3OH), *m/z* [M + Ph_3Sn]$^+$.

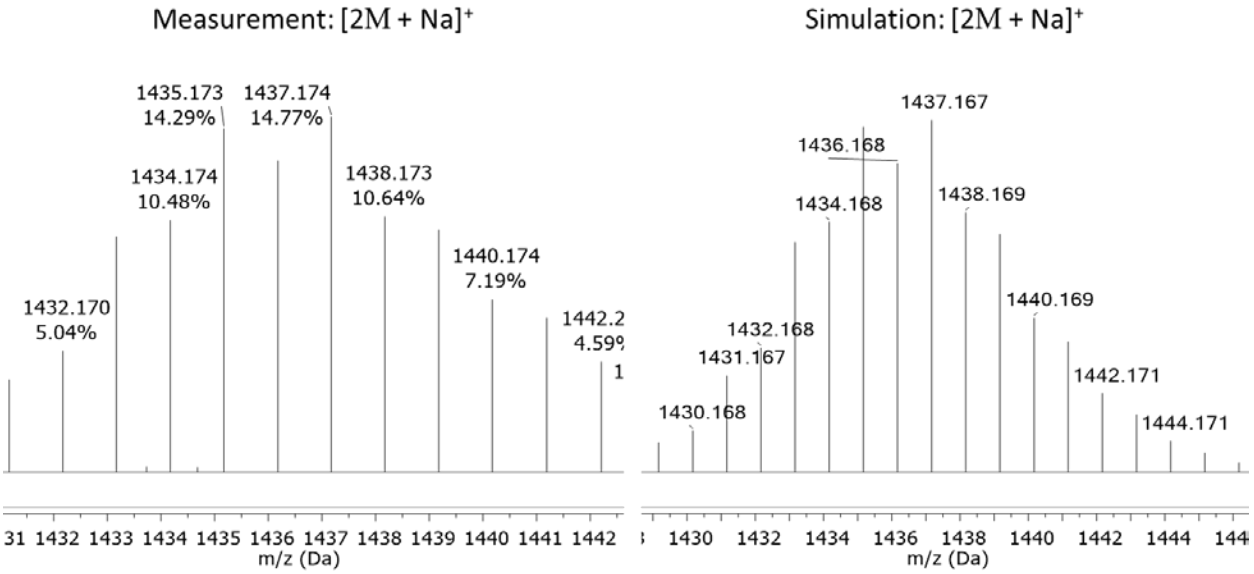

Figure S5. HR-ESI-MS (positive mode, CH_3OH), m/z [2M + Na]$^+$.

HR-ESI-MS (positive mode, CH_3OH): *m/z* [M+Ph_3Sn]$^+$: calcd. for $C_{55}H_{45}ClNO_4Sn_2$: 1056.108, found: 1056.134; *m/z* [2M+Na]$^+$: calcd. for $C_{74}H_{60}Cl_2N_2O_8Sn_2Na$: 1437.167, found: 1437.174; the observed isotopic pattern is in agreement with the calculated one.

X-ray Crystallography

Table S1. Crystal data and structure refinement of $[Ph_3Sn(IND)]$

Empirical Formula	$C_{39.50}H_{32.50}Cl_{8.50}NO_4Sn$
Molecular Formula	$[C_{37}H_{30}ClNO_4Sn] \cdot 2.5\ CHCl_3$
Formula weight [g mol^{-1}]	1005.18
T [K]	130(2)
Crystal system	Tetragonal
Space group	$I4_1/a$
Unit cell dimensions	
a [Å]	31.6697(7)
b [Å]	31.6697(7)
c [Å]	16.8632(5)
α [°]	90
β [°]	90
γ [°]	90
Volume [Å^3]	16913.3(9)
Z	16
$\rho_{(calc.)}$ [Mg m^{-3}]	1.579
μ [mm^{-1}]	1.182
F(000)	8048
Crystal size [mm^3]	0.40 x 0.10 x 0.05
Θ_{min}– Θ_{max} [°]	1.819 – 30.421
Index ranges	$-44 \leq h \leq 42$
	$-42 \leq k \leq 39$
	$-23 \leq l \leq 23$
Reflections collected	81150
Independent reflections [$R_{(int)}$]	11803 [0.0899]
Completeness (Θ [°])	100.0% (28.285)
T_{Max} / T_{Min}	1.00000 / 0.53444
Data / restraints / parameters	11803 / 381 / 732
Goof [on F^2]	1.045
*R*1, w*R*2 [I>2 σ (I)]	0.0607, 0.1520
*R*1, w*R*2 (all data)	0.1165, 0.1837
Residual electron density [e·Å^{-3}]	1.269 / -0.713
CCDC deposition number	2226528

NMR Spectra of [$Ph_3Sn(FBP)$]

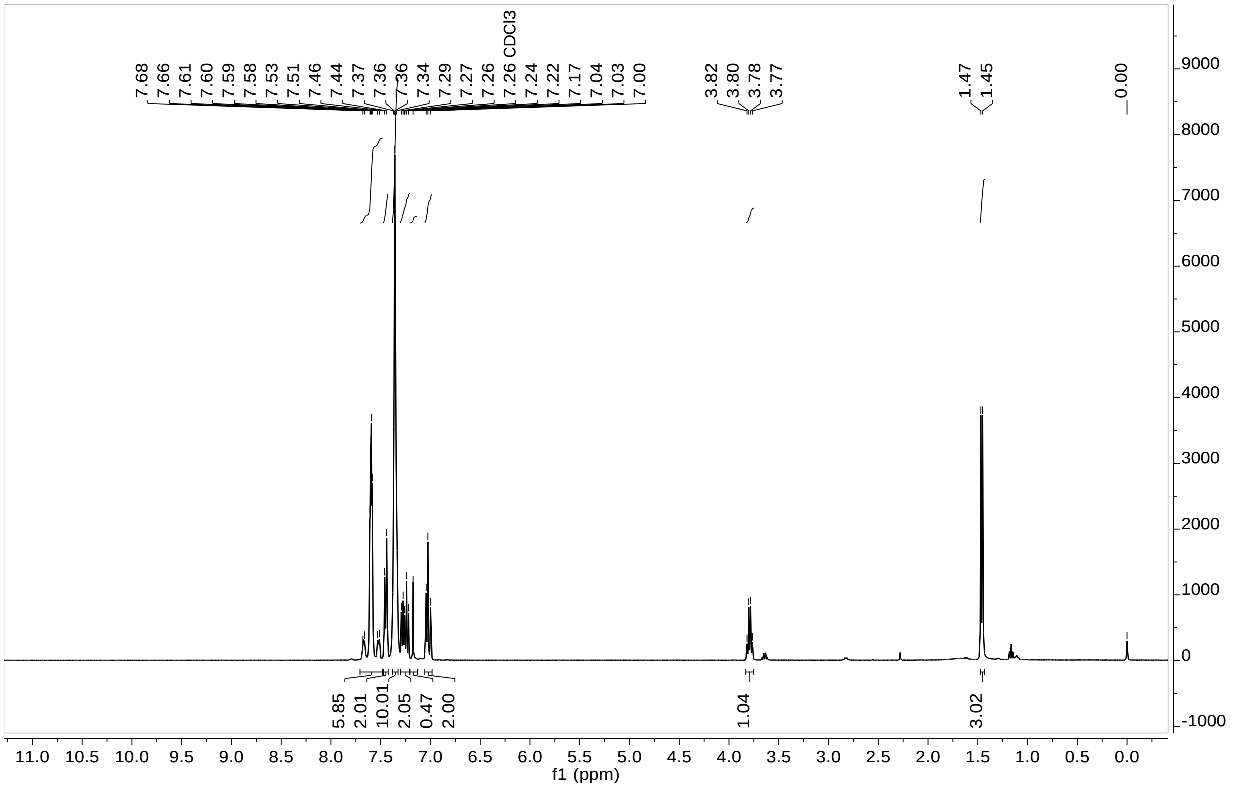

Figure S6. ^{1}H NMR spectrum of [$Ph_3Sn(FBP)$] in $CDCl_3$.

^{1}H NMR ($CDCl_3$, ppm, 400 MHz): δ = 7.68 – 7.51 (m, br., 6H, CH_{aryl}), 7.45 (d, $^{3}J_{HH}$ = 8 Hz, 2H, CH_{aryl}), 7.37 – 7.34 (m, br., 10H, CH_{aryl}), 7.28 (d, $^{3}J_{HH}$ = 8 Hz, 1H, CH_{aryl}), 7.24 (t, $^{3}J_{HH}$ = 8 Hz, 1H, CH_{aryl}), 7.17 (s, 1H, CH_{aryl}), 7.03 (t, $^{3}J_{HH}$ = 8 Hz, 2H, CH_{aryl}), 3.79 (q, $^{3}J_{HH}$ = 8 Hz, 1H, CH), 1.46 (d, $^{3}J_{HH}$ = 8 Hz, 3H, CH_3).

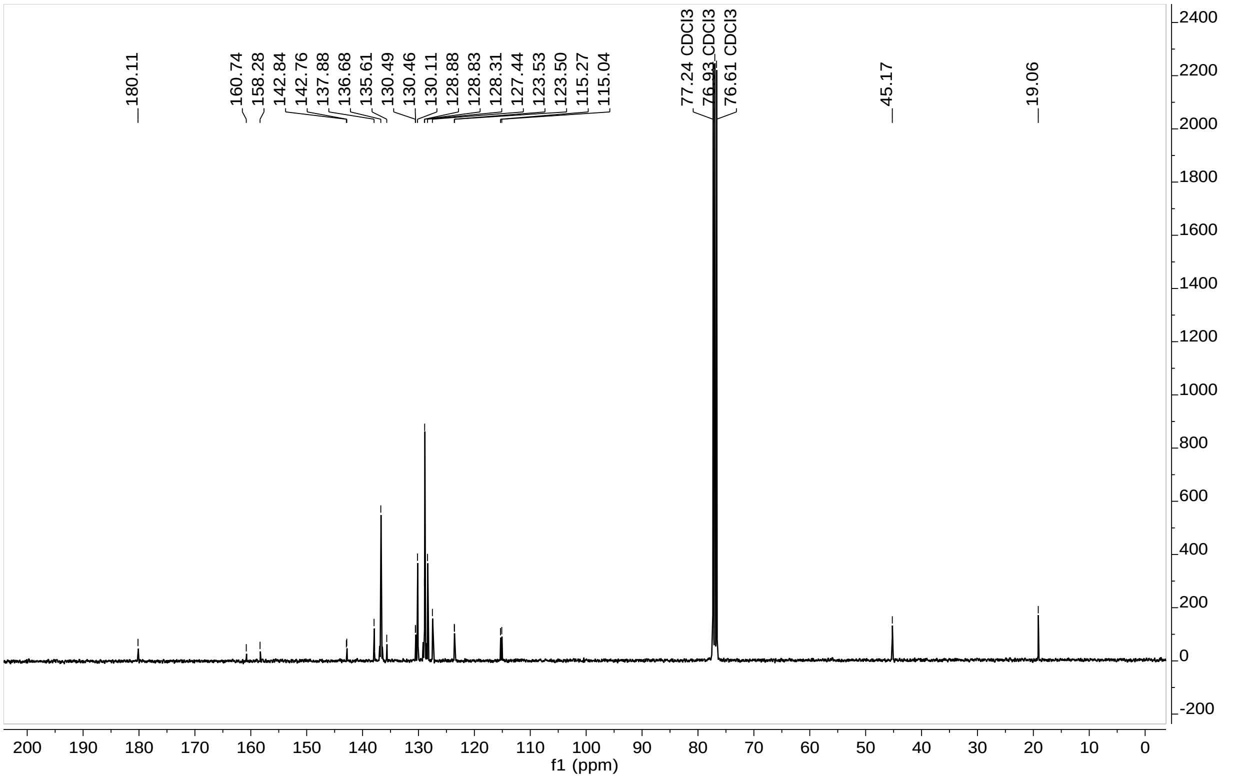

Figure S7. $^{13}C\{^1H\}$ NMR spectrum of [Ph_3Sn(FBP)] in $CDCl_3$.

$^{13}C\{^1H\}$ NMR ($CDCl_3$, ppm, 100.6 MHz): δ = 180.1 (qC, *C*OOH), 160.7 (qC, C_{aryl}), 158.3 (qC, C_{aryl}), 142.8 (qC, C_{aryl}), 137.9 (CH, C_{aryl}), 136.7 (CH, C_{aryl}), 135.6 (qC, C_{aryl}), 130.5 (qC, C_{aryl}), 130.1 (CH, C_{aryl}), 128.9 (CH, C_{aromat}), 128.3 (CH, C_{aryl}), 127.4 (CH, C_{aryl}), 123.5 (CH, C_{aryl}), 115.3 (CH, C_{aryl}), 115.0 (CH, C_{aryl}), 45.2 (*C*H), 19.1 (*C*H_3).

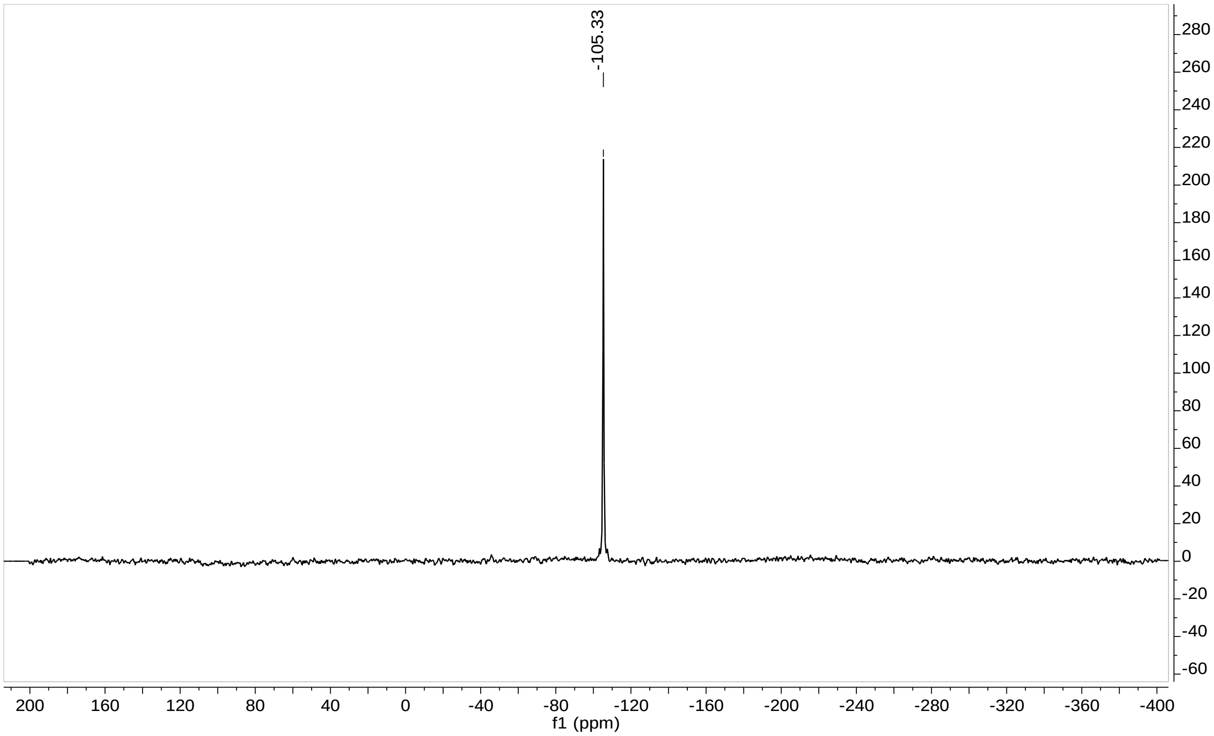

Figure S8. $^{119}Sn\{^1H\}$ NMR spectrum of $[Ph_3Sn(FBP)]$ in $CDCl_3$.

^{119}Sn{^{1}H} NMR ($CDCl_3$, ppm, 149.2 MHz): δ = -105.33.

Mass Spectra of [$Ph_3Sn(FBP)$]

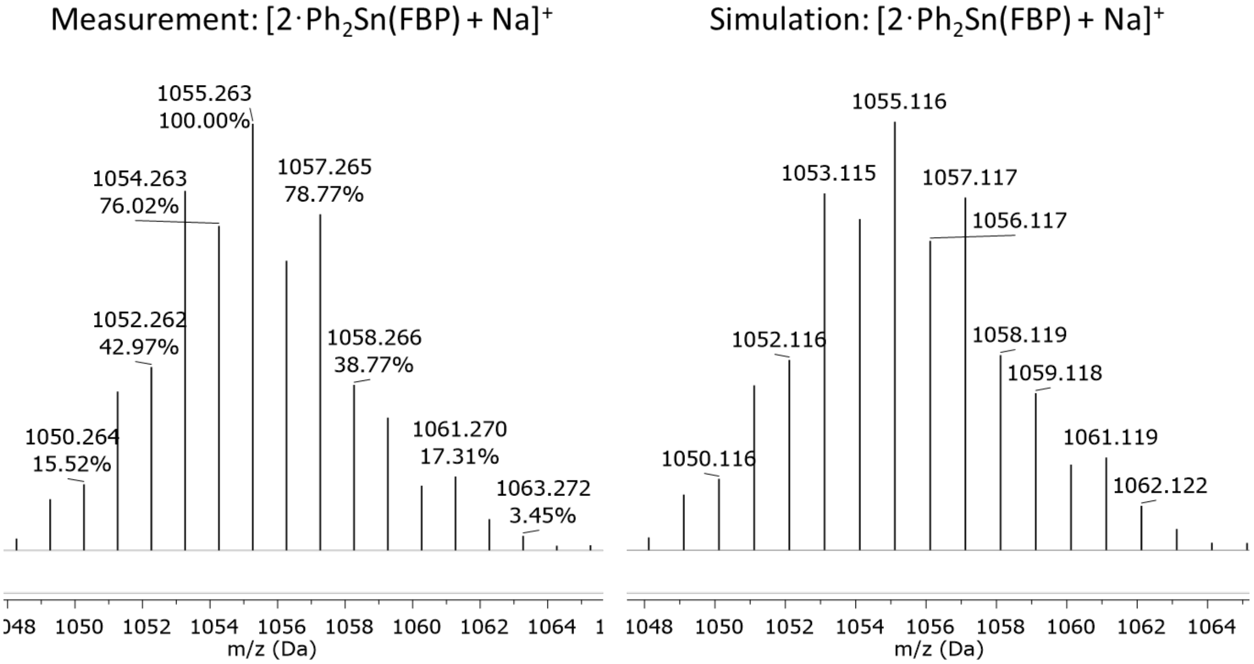

Figure S9. HR-ESI-MS (positive mode, CH_3OH), *m/z* [2·$Ph_2Sn(FBP)$ + Na]$^+$.

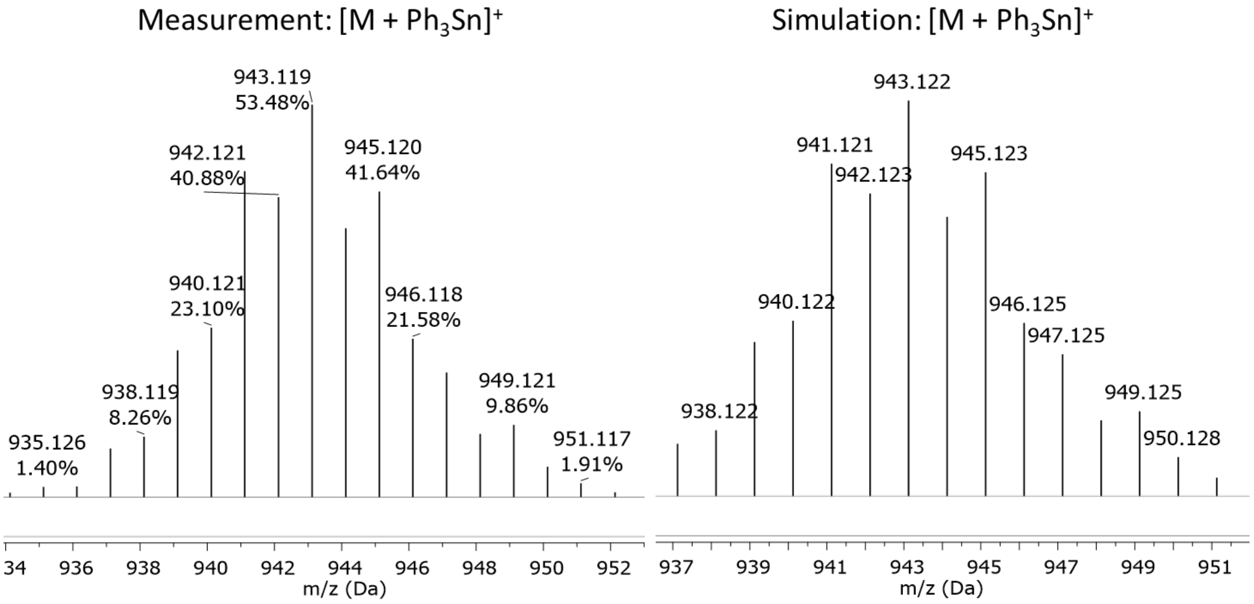

Figure S10. HR-ESI-MS (positive mode, CH_3OH), *m/z* [M + Ph_3Sn]$^+$.

HR-ESI-MS (positive mode, CH_3OH): *m/z* [2·$Ph_2Sn(FBP)$+Na]$^+$: calcd. for $C_{54}H_{44}F_2O_4Sn_2$: 1055.116, found: 1055.263; *m/z* [M+Ph_3Sn]$^+$: calcd. for $C_{51}H_{42}FO_2Sn_2$: 943.122, found: 943.119; the observed isotopic pattern is in agreement with the calculated one.

Stability of complexes [Ph_3Sn(IND)] and [Ph_3Sn(FBP)] in DMSO

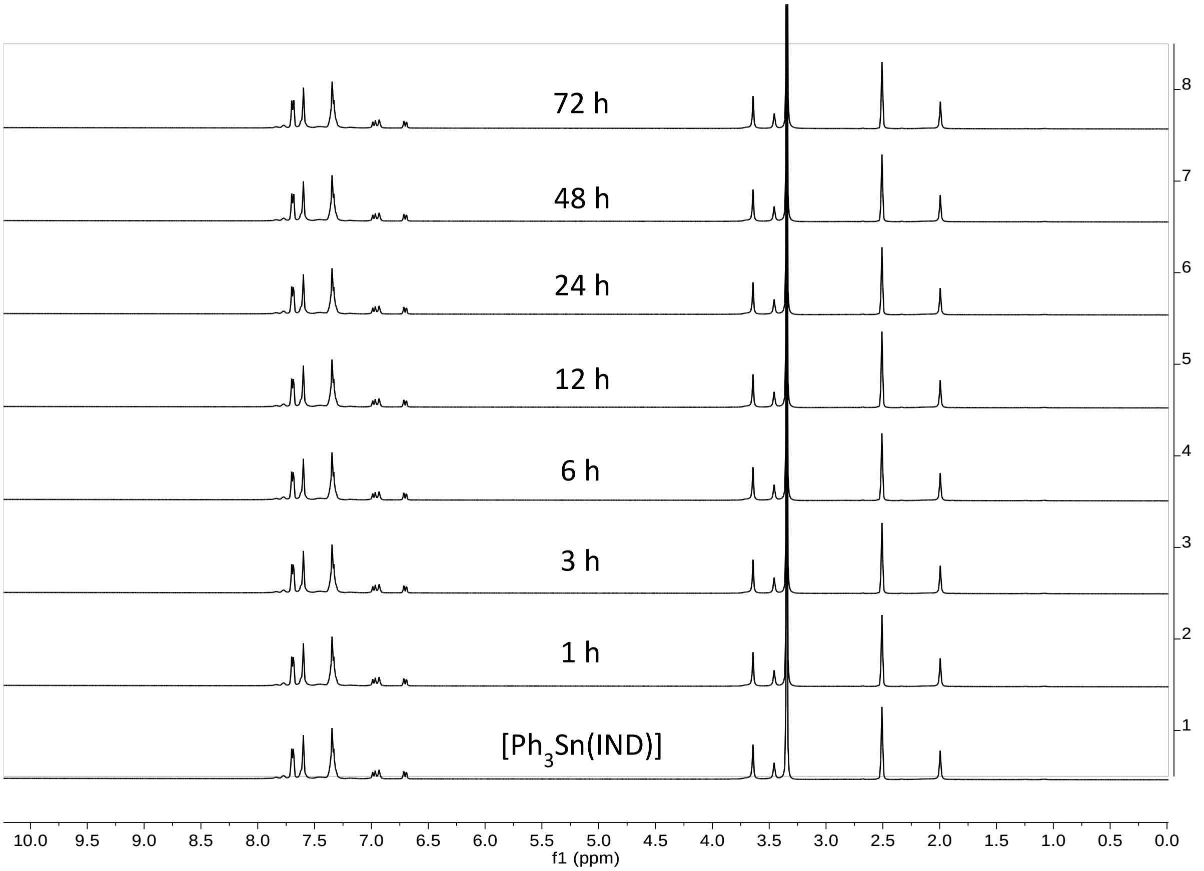

Figure S11. Stability of [Ph_3Sn(IND)] in DMSO-d_6 over 72 h; time-resolved ^1H NMR spectra.

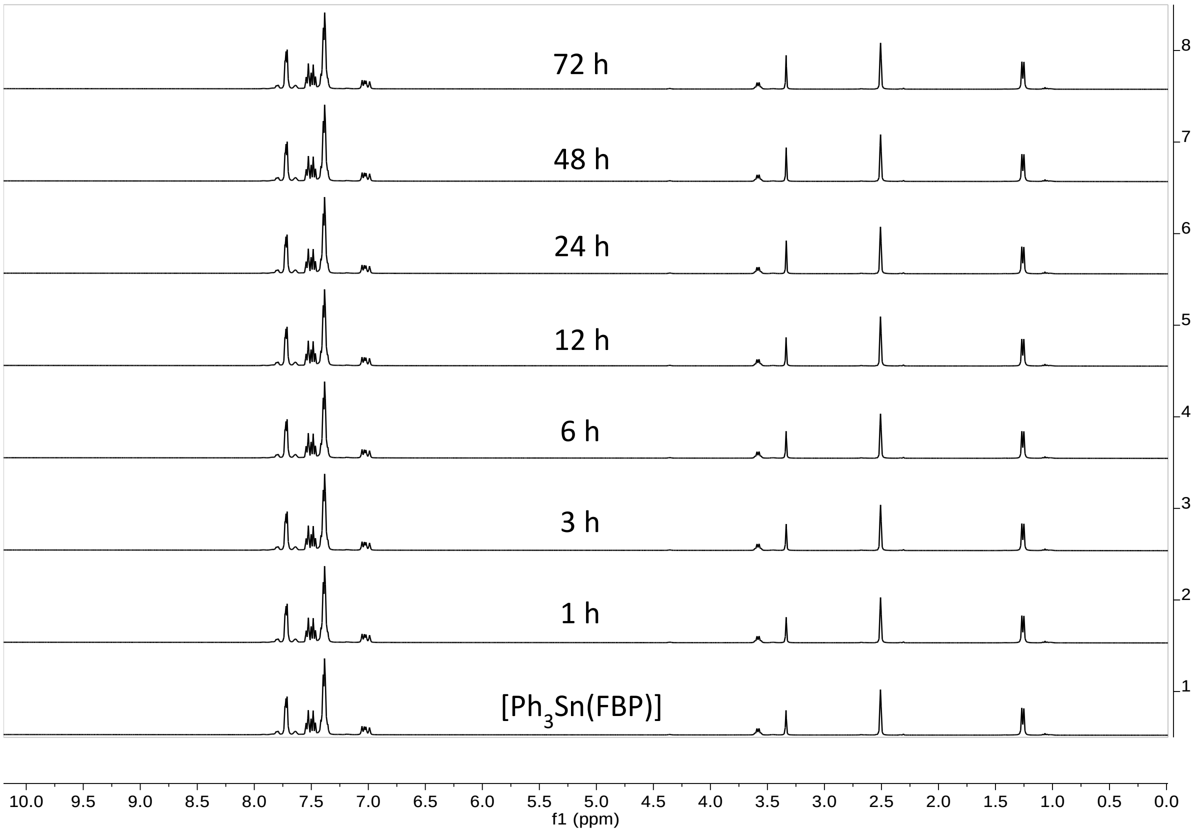

Figure S12. Stability of [Ph_3Sn(FBP)] in DMSO-d_6 over 72 h; time-resolved ^{1}H NMR spectra.

Cell viability of complexes

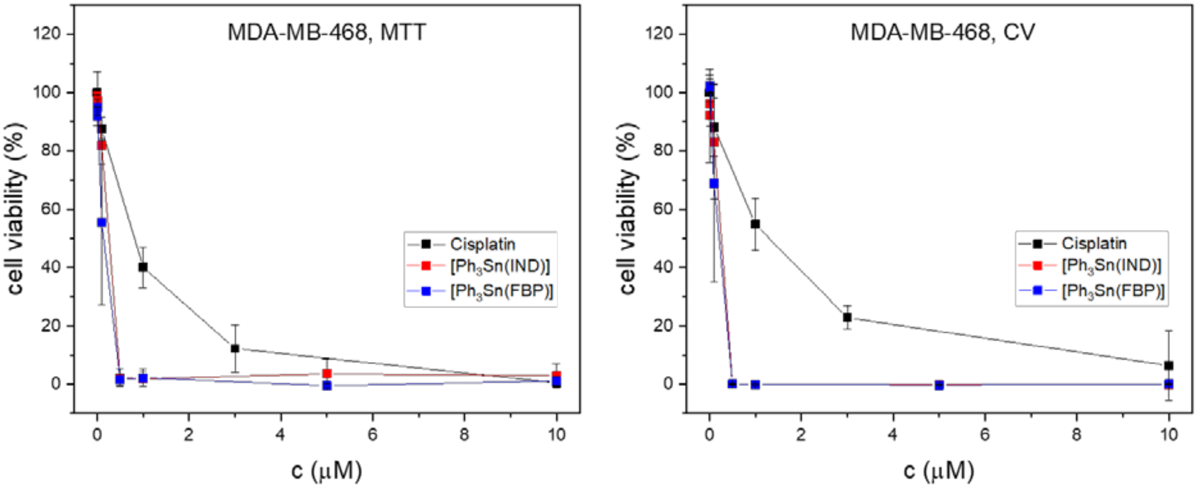

Figure S13. Cell viability of [Ph_3Sn(IND)], [Ph_3Sn(FBP)] and cisplatin determined by MTT and CV assays in MDA-MB-468 breast cancer cell line.

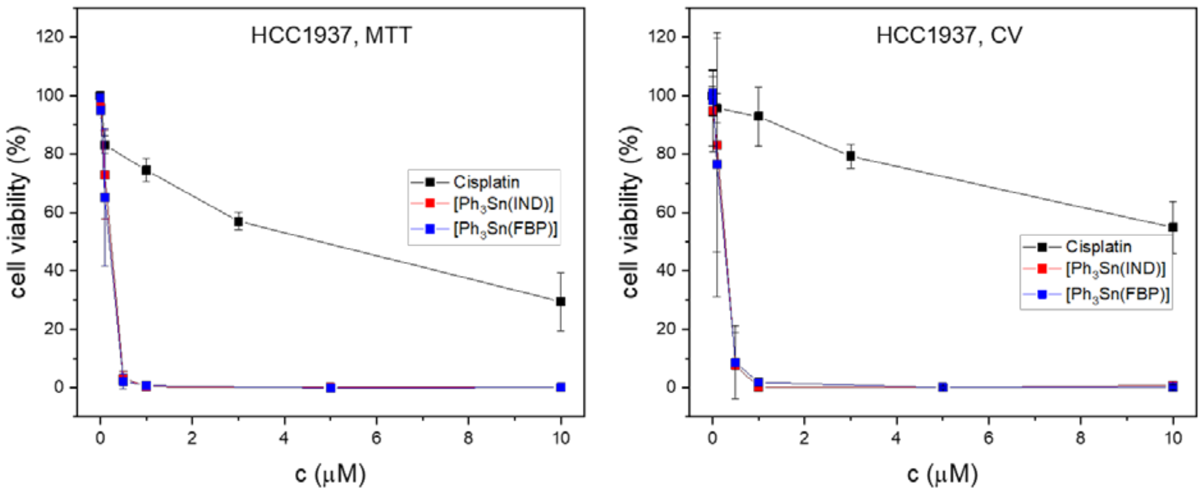

Figure S14. Cell viability of [Ph_3Sn(IND)], [Ph_3Sn(FBP)] and cisplatin determined by MTT and CV assays in HCC1937 breast cancer cell line.

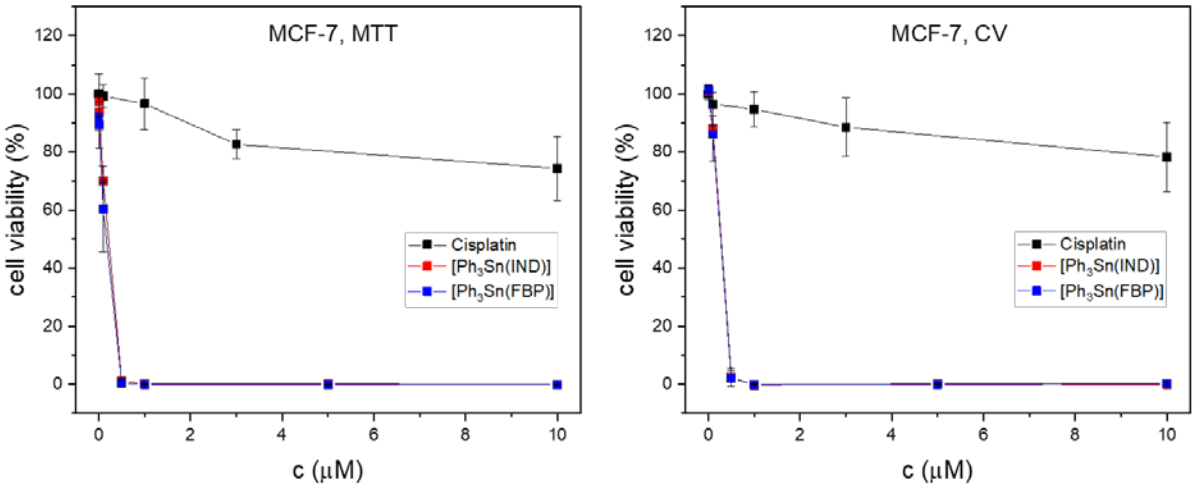

Figure S15. Cell viability of [Ph_3Sn(IND)], [Ph_3Sn(FBP)] and cisplatin determined by MTT and CV assays in MCF-7 breast cancer cell line.

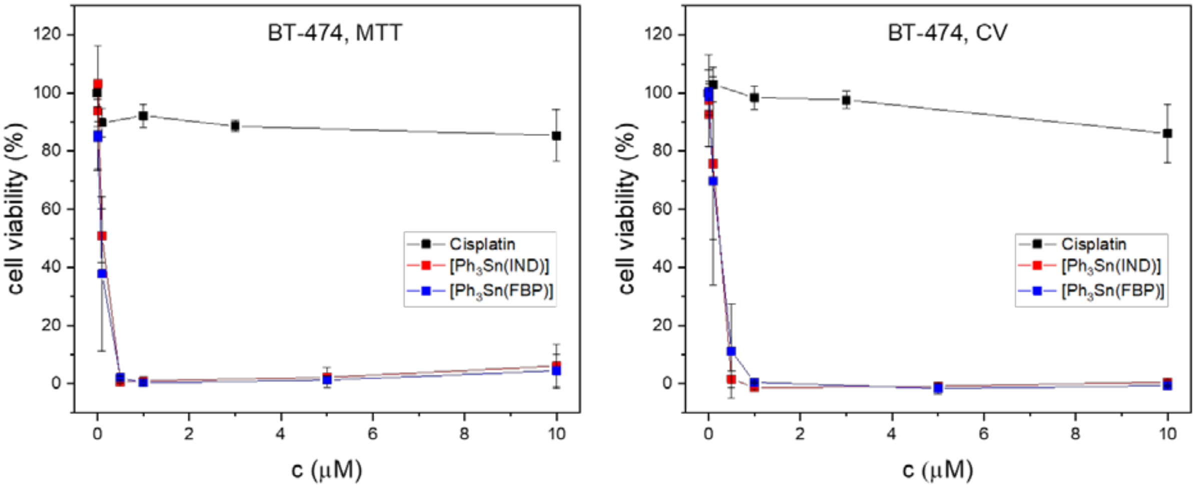

Figure S16. Cell viability of [Ph_3Sn(IND)], [Ph_3Sn(FBP)] and cisplatin determined by MTT and CV assays in BT-474 breast cancer cell line.
